# Ethical dilemmas embedded in performing fieldwork with nurses in the ICU

**DOI:** 10.1177/0969733021996025

**Published:** 2021-04-08

**Authors:** Monica Evelyn Kvande, Charlotte Delmar, Jette Lauritzen, Janne Brammer Damsgaard

**Affiliations:** 155319 Lovisenberg Diaconal University College, Norway; 1006 Aarhus University, Denmark; VID Bergen, Norway; 317905 VIA University College, Denmark; Aarhus University, Denmark; 1006 Aarhus University, Denmark

**Keywords:** Ethics of care/care ethics, fieldwork, intensive care, qualitative research, research ethics, researcher–participant relationship

## Abstract

**Background:**

Background: In general, qualitative research design often involves merging together various data collection strategies, and researcher’s may need to be prepared to spend longer periods in the field to pursue data collection opportunities that were not foreseen. Furthermore, nurse researchers performing qualitative research among patients and their relatives often experience unforeseen ethical dilemmas.

**Aim:**

This paper aimed to explore aspects of ethical dilemmas related to qualitative nursing research among patients and their relatives in the intensive care unit (ICU).

**Research design:**

This paper is based on a qualitative researcher’s personal experience during a hermeneutic phenomenological study involving close observation and in-depth interviews with 11 intensive care nurses. Data were collected at two ICUs in two Norwegian university hospitals.

**Ethical considerations:**

The study was approved by the Norwegian Social Science Data Services (NSD). The Regional Committee for Medical and Health Research Ethics (REK) granted dispensation to the project regarding health personnels confidentiality of the patients who were present during the observation (2012/622-4).

**Findings:**

Close observation with nurses in the ICU requires the researcher to balance being a qualitative researcher, an ICU nurse and a sensitive fellow human being open to the suffering of the other—that is, being embodied, engaged and affected by sensitive situations and simultaneously constantly stepping back and reflecting on the meaning of those situations.

**Conclusions:**

The qualitative researcher’s ethical awareness also entails knowing and acknowledging his or her own vulnerability, which becomes apparent in the researcher-participant relationship and settings in which being a fellow human always overrules the researcher’s role in ethical dilemmas.

## Introduction

The purpose of this article was to explore aspects of ethical dilemmas related to qualitative nursing research among patients and their relatives in the intensive care unit (ICU).

In general, qualitative research design often involves merging together various data collection strategies, and researchers may need to be prepared to spend longer periods in the field to pursue data collection opportunities that were not foreseen.^1^ Therefore, a nursing researcher performing field work in a familiar area of nursing must set clear boundaries between oneself, the patients, and their family as well as other staff through a continuous process of reflexivity about the emic and ethic observations and interaction with the informants. Otherwise, these boundaries can become obscured and the role as a nurse researcher can become difficult to uphold.^2,3^

The motivation for this article is our own experiences both as researchers and nurses.^4–6^ Based on fieldnotes from a hermeneutic phenomenological study,^7^ we discuss “ethical dilemmas embedded in the meeting between a researcher, the patient and their relatives when doing fieldwork in the ICU.” Benner et al.^8^ and Brinkmann and Kvale^9^ argue that particular exemplars of ethically justifiable and ethically questionable research can add depth, nuances, and qualitative distinctions and can contribute to ethical reflections. Furthermore, Brinkmann and Kvale^9^ state that examples help researchers evaluate their research practice and point to ethical descriptions of situations as an approach to learning ethical behavior in qualitative research.

## Background

In general, qualitative researchers often combine a complex array of data derived from a variety of sources and use a variety of methods.^1^ One such method is close observation. According to van Manen,^10^ close observation generates different forms of material than those that can be obtained via written or interview approaches. During close observation, the researcher is present when incidents occur: this proximity gives the researcher firsthand knowledge of anecdotes relevant for his or her research project.^10,11^ Furthermore, van Manen^10,11^ explained that the method of close observation requires that one simultaneously be both a participant and an observer and involves an attitude of assuming a relationship that is as close as possible while retaining a hermeneutic alertness to situations that allow us to constantly step back and reflect on the meaning of these situations.

The empirical basis for our ethical discussion is personal experience as a researcher during a hermeneutic phenomenological study of the phenomenon of becoming aware of signs of incipient changes in patients’ clinical conditions, including both improvement and deterioration.^5,12^ Data were collected through close observations of bedside nursing and in-depth interviews with 11 ICU nurses during a 10-month period spanning from December 2012 to September 2013. Field notes were written in a notebook during the observations, and more detailed descriptions of the observations were recorded immediately after each shift.

Intensive care patients have life-threatening conditions and require life-sustaining interventions and technological support for survival, which entails continuous monitoring of their vital functions, dynamic interventions, and health-promoting activities.^13^ In the ICU, patients can experience discomfort, loss of control, transformations of perception, and surreal experiences.^14,15^ The admission of a family member to the ICU in a life-threatening condition places heavy stress and anxiety on a family in addition to the uncertainty and fear involved with potentially losing a family member.^16,17^

Close observation of intensive care nurses in ICUs implies that patients and their relatives become indirectly involved parties. The Regional Committee for Medical and Health Research Ethics (REK) granted dispensation to the project regarding health personnels’ confidentiality of the patients who were present during the observation period (2012/622-4). Based on recommendations made by the REK, the patients’ relatives received written and oral information about the research project and their right to make requests for close observations on behalf of the patient.

Researchers are guided by ethical principles to protect study participants, and they must ensure that their studies are based on justice, beneficence, and respect for human dignity.^1^ In addition, the researcher must understand procedures such as risk and benefit assessments, informed consent, confidentiality, and treatment of vulnerable groups.^1^ Clinical research involving critically ill patients is necessary to improve their care and outcomes. However, critically ill patients are often unable to provide informed consent for research due to their illness or treatment.^18,19^ Permission to conduct research among critically ill patients may present a dilemma because patients who are eligible to participate in research are often unable to provide consent, and their healthcare proxies are either unavailable or overwhelmed.^20^ Thus, researchers have relied on different frameworks to obtain consent in the ICU, most commonly using substitute decision makers.^19^

In the following, we present the personal experience of a researcher when performing fieldwork with nurses in the ICU. According to van Manen,^10^ presenting a personal description of a lived experience involves describing one’s experience as much as possible in experiential terms while focusing on a particular situation. Furthermore, a personal experience, called a “lived-experience description,” is a description of the experience, as it was, that involves one’s state of mind, including feelings, mood, and emotions.

## “Close observation of ICU nurses—a balancing act”

It is morning, and I [researcher] am together with Jenny [the nurse], who is caring for an adult mechanically ventilated patient with a serious condition who is in a life-threatening situation.

When I enter the ICU room, I feel overwhelmed. There are three patients in the room, three patients with different disorders, and each of them seems to be in the same bad condition. Monitors, infusion pumps, ventilators, and dialysis machines are scattered around the patient’s bed, each of which generates a high sound level. Alarms frequently indicate that something is wrong and must be adjusted. I can hear the rhythmic sound of the ventilator as an ongoing hum. There are many healthcare workers in the ICU room, and I can see 1–2 nurses at the bedside of each of the three patients.

Jenny’s priority immediately after the report from the night shift is to start morning care. I am assisting Jenny in morning care of the patient, and I can see that she is working in a concentrated and systematic manner at the bedside. Morning care is a bit split up because she [the patient] is so unstable and demanding in connection with oral hygiene. I can see that Jenny has a worried expression on her face, and she often stops and asks, “What happens now?” Jenny did not get a thorough-enough initial observation of the patient and says,

I did not make these observations right away, so I have to do them after the situation is calm…I think it is important that I know what I have in terms of initial observations so that I have something to relate it to if changes occur, and changes do normally occur.

In the visiting hour, the patient’s father comes to the ICU. I can see in his body language that the patient’s serious and unstable condition places heavy stress and anxiety on him. He is tense in his body, pale in the face, tears tremble, and I can see desperation in his eyes. I feel so sorry for him and it simply got under my skin, and it is difficult to focus on the work of Jenny.

The patient’s father addressed me as a nurse with questions about the patient’s condition, treatment plan, and prognosis in the same manner that he addressed Jenny. I experience that as an ethical dilemma, and I feel that it is difficult to balance being an ICU nurse with limited competence and at the same time not dismiss the patient’s father’s appeals for help. I place my hand on his shoulder and say, “I can understand that you have a lot of questions, and I will tell the ICU nurse so that she can inform you.” I can see in his eyes that he is insecure, and I am thinking that he may perceive that I know something I don’t want to tell him.

I feel that I need to keep my distance and feel that it is far too much for me, and I feel vulnerable and overwhelmed by my own feelings. I agree with Jenny that I will go to a quiet place outside the ICU and write field notes in a notebook based on what I sense (sight, hearing, smell, and touch) and emotions.

When I am back, I continue to assist Jenny in nursing care during the shift, for example, oral hygiene, changing the position in bed, and tracheal suction. At the end of the shift, the patient’s condition is more stable, and the situation is more calm (Fieldnote, 2012).

## Ethical dilemmas embedded in the experience description

In the close observation of Jenny, I encountered a situation that affected me as a researcher, an ICU nurse and a human being. I was touched and moved by meeting both a patient who was in a critical and life-threatening situation and her father.

It was also a balancing act between assisting Jenny in nursing care within the limits of my competence [as an ICU nurse] and what was ethical for me as a researcher to participate in. I also wondered how that affected the observations.

The analyses of the fieldnote were performed using the reflective methods of van Manen^10,11^ including thematic reflections. Thematic reflection refers to the process of recovering structures of meaning that are embodied in the researcher’s experience, as represented in text. Grasping and formulating a thematic understanding is a complex and creative process; it is not a rule-bound process but is instead a free act of “seeing” meaning.

The personal experience of the researcher describes the following aspects of ethical dilemmas experienced when performing fieldwork with nurses in the ICU:

− Being a qualitative researcher, an ICU nurse, and a fellow human being open to the suffering of the other.
− Being a participant and at the same time an observer; being embodied, engaged, and affected by sensitive situations and simultaneously constantly stepping back and reflecting on the meaning of those situations.

These findings led us to the phenomenology of sensation of the Danish philosopher Knud Ejler Løgstrup, the thinking of the Norwegian nurse and philosopher Kari Martinsen and the thinking of Løgstrup in a clinical nursing context. Their perspectives were used to reflect on the themes with the goal of arriving at a comprehensive understanding of the researcher’s experience when performing fieldwork with nurses in the ICU.

## Discussion

### Being open to the suffering of the other

The personal description reflects ethical dilemmas and demonstrates the challenge of carefully balancing being a qualitative researcher, an intensive care nurse, and a fellow human being and witnessing the suffering of the other.^21^ Martinsen^22^ explains that when nurses in a clinical context are sensitive and attentive, they are receptive, touched, and moved to respond to the unique patient’s appeal and needs.

People who are or who become recipients of healthcare in general and nursing care in particular can be considered vulnerable. In the context of nursing practice, patients are not always able to either make judgments about self-protective actions or to know when they are being exploited.^23^ Patient vulnerability is a key concept in nursing, and protecting the patient from harm is a fundamental part of the role of nurses.^23,24^ Sellman^23^ defines the vulnerability of severely ill patients’ vulnerability using the term “more-than-ordinary.” Furthermore, for unconscious patients in the ICU, nurses’ competence to provide care depends on the nurses’ capacity to recognize the specific and general vulnerability of a given individual and to act in suitably protective ways. In relation to the personal description, the researcher’s sensitivity when meeting the patient and her father was a core nursing attribute. The researcher tried to understand their situation and not dismiss the patient’s father’s appeals for help. Purdy^25^ explains that the essence of being vulnerable is openness to circumstances and the foundation of being influenced. In relation to the personal description, this statement means that in close observation of ICU nurses, the researcher, who was also an experienced ICU nurse, was touched and moved by interacting with both the critically ill patient in a life-threatening situation and her father. During the moments of openness and attentiveness described in the experience description, the researcher may have been vulnerable. This observation is similar to that of Angel and Vatne,^24^ who explain that nurses’ vulnerability lies in their engagement in caring for patients. This observation may also be congruent with the findings of Angel and Vatne,^26^ who demonstrated that by being open and sensitive to patients and their needs, nurses may experience their own sense of vulnerability, which may manifest in feelings of being out of control and overwhelmed by emotions. The researcher in the personal description was open to the suffering of the patient and her father and was sensitive to what they expressed through their bodies. The researcher felt overwhelmed by the situation and her own strong emotions and caused emotions of unease in others; thus, the researcher found it difficult to focus on the work of Jenny.

Haahr et al.^27^ explain that the researcher’s ethical awareness also entails knowing and acknowledging his or her vulnerability, which becomes apparent in the researcher–participant relationship. In difficult interview settings, being a human always overrules the researcher role in ethical dilemmas. In relation to the personal description, this may mean that although a situation is meaningful from a research perspective, a researcher leaves when she or he interprets her presence as burdensome for the nurse, the patient, and the patient’s relatives. In this particular experience, the researcher felt that it was not ethical to participate in the situation when the patient’s father was visiting the patient. The researcher sensed that the father became uneasy because of her presence and considered that the right thing to do was to leave the ICU room. This observation may be similar to the findings of Clancy,^28^ in which the researchers are reminded of the vulnerability of all persons in the research project and give rise to legitimate questions such as “Is my presence here right now justified?” even though issues of informed consent have been addressed.

Martinsen^29^ explains that the mind is in sensation, always touched and moved by the situation, and that to receive an impression is to be sensitively moved. In relation to the researchers’ personal experience, this statement means that the researcher is receptive to the suffering of the other such as through eye contact, facial expression, body movement, and anxiety. The researcher felt the suffering of the patient and her father in such a strong way that the researcher became affected by it and felt vulnerable. This is similar to the findings of Thorup et al.,^30^ who demonstrated that vulnerability and suffering have been proven to be sensitive issues for nurses, like a sore point that either serves as an eye-opener by contributing to a deeper understanding of the patients’ vulnerability or causes the development of blind spots.

### Being a participant and an observer

The method of close observation requires that one simultaneously is a participant and an observer.^10,11^ In relation to the personal description, this statement means that the researcher participated in nursing care and at the same time closely observed Jenny’s everyday practices and interactions with the patient; thus, the researcher was embodied, engaged, and affected by sensitive situations and at the same time constantly stepping back and reflecting on the meaning of those situations.

Close observation enabled the researcher to focus on the meaning expressed by an individual nurse’s entire body, such as gestures, facial expressions, and posture, which changed with changes in the patient’s condition. If the patient’s condition became unstable or unclear, Jenny moved closer to the patient’s bedside and started to examine the patient using her senses (sight, hearing, smell, and touch) and by monitoring. Løgstrup^31^ explains that our understanding of other humans is an understanding of impressions of them and not their characteristics. This impression gives us access to them, and mimicry, gestures, tone, and body posture play a crucial role. Similarly, Van der Meide et al.^32^ explain that both articulated speech and nonarticulated speech—body language—are considered valid ways of expressing meaning.

In practice, this means that close observation involves a balancing act, that is, drawing as close as possible to Jenny’s way of being and experiences, including the situations and settings in which she is involved, while distancing or retaining a hermeneutic alertness as an ongoing process in which the researcher takes a step back and reflects on the meaning of various situations and episodes. This is similar to Høgh et al.,^33^ who demonstrated that the role as a researcher when performing field observations was a continuous balance between intimacy and distance, being personally engaged, preserving dignity and confidence, and at the same time maintaining an analytic distance. Løgstrup^31^ explained that without distance, we would be lost in sensation and unable to understand the situation. The researcher in the experience description was engaged and affected by sensitive situations during the shift and wondered how this could have affected the observation. The situation was overwhelming, and the researcher felt the need to maintain distance to focus on observing the nurse. Martinsen^22^ explained the notion of life interpretation (*tydning*) as a way for nurses to be present in a situation. Clarification requires nurses to be receptive, attuned, and attentively present in encounters with patients and not to remain outside what should be clarified. Clarification involves being in the shifting interplay between sensation and understanding, searching for words that may help to clarify meanings in impressions. The researcher created distance by “taking a step back” to a quiet place outside the ICU and thus attempted to understand what was sensed in relation to the unique situation. Løgstrup^34,35^ explained that understanding creates both distance between the sensed and the sensing and an open space in which to move and think.

Emotions and thoughts that were evoked by what the researcher sensed during the observation were written in a notebook which enabled meaningful insights. Løgstrup^31,35,36^ stated that with language, understanding creates distance between the sensed and the sensing and creates an open space in which to move and think. In this space, which Løgstrup^35,36^ calls “the fictive space of understanding,” sensation reaches into understanding and makes it intuitive.

## Conclusion

Ethical dilemmas when conducting close observation with nurses in the ICU are far more complex than the researcher might anticipate.

Close observation with nurses in the ICU requires the researcher to balance being a qualitative researcher, an ICU nurse, and a sensitive fellow human being open to the suffering of the other—that is, being simultaneously a participant and an observer.

The qualitative researcher’s ethical awareness also entails knowing and acknowledging his or her own vulnerability, which becomes apparent in the researcher–participant relationship and settings in which being a fellow human always overrules the researcher’s role in ethical dilemmas.

Knowledge about how to act ethically when conducting qualitative nursing research among vulnerable patients and settings is a subject for future research education in relation to ethical matters.
